# Mendelian randomization revealed a one-way causal association between increased Isovalerylcarnitine (C5) levels and the risk of idiopathic pulmonary fibrosis

**DOI:** 10.1097/MD.0000000000043555

**Published:** 2025-08-08

**Authors:** Jing He, Zhengyue Liao, Hongyu Chen, Jiaojiao Fu, Sijing Liu, Ya’nan Hua, Jinlin Guo

**Affiliations:** a Department of Medical Laboratory, Affiliated Guang’an District People’s Hospital of North Sichuan Medical College, Guang’an, China; b College of Medical Technology, Chengdu University of Traditional Chinese Medicine, Chengdu, China; c Chongqing Key Laboratory of Sichuan-Chongqing Co-construction for Diagnosis and Treatment of Infectious Diseases Integrated Traditional Chinese and Western Medicine, Chengdu, China; d School of Transportation and Logistics, Southwest Jiaotong University, Chengdu, China.

**Keywords:** fatty acid metabolism, IPF, Isovalerylcarnitine (C5) levels, SNPs, two-sample MR

## Abstract

There have been multiple observational studies that have established a link between metabolite levels in the body and idiopathic pulmonary fibrosis (IPF), specifically focusing on metabolites derived from fatty acids. However, a complete understanding of the precise molecular and biological factors, as well as the causality between them, remains elusive. The main objective of our study was to evaluate the potential causal relationship between blood metabolites and IPF by using Mendelian randomization (MR). To achieve this goal, we utilized the most comprehensive genome-wide association study to date, which identified genetic variants associated with blood metabolites (1091 blood metabolites and 309 metabolite ratios). Summary statistics of IPF were collected from Finngen R8 (1812 IPF patients and 338,784 controls), inverse variance weighted method (IVW) is used as the main method in determining causality. Isovalerylcarnitine (C5) levels (OR = 1.2435, 95% CI: 1.0494–1.4736, *P*val = .0119) was found significantly related to higher risk of IPF. There was no significant heterogeneity in our study (IVW method: *P*val = .132; MR-Egger method: *P*val = .105) and horizontal pleiotropy (β = ‐0.027; SE = 0.0337; *P*val = .4310). The sensitivity analysis did not reveal any potential abnormal drivers (0.1 < All < 0.3). Two-sample MR method demonstrated the causal relationship between blood metabolites and IPF, and further studies found that Isovalerylcarnitine (C5) levels, as a potential biological risk factor for IPF, may provide a new target for the treatment of IPF.

## 1. Introduction

Idiopathic pulmonary fibrosis (IPF) is a progressive and irreversible primary interstitial lung disease characterized by pulmonary inflammation and fibrotic changes.^[1]^ Despite extensive research, the exact cause of IPF remains unknown. Epidemiological data indicates that the prevalence of IPF in Europe ranges from 3 to 9 cases per 100,000 people.^[2]^ Subsequent data revealed that the incidence of IPF has increased by 4%, with a strong correlation between age and incidence.^[3]^ For terminal patients, lung transplantation is often considered as a management strategy.^[4]^ Recent studies have suggested that small lipid molecules, such as fatty acids, cholesterol, arachidonic acid metabolites, and phospholipids, may play a significant role in the development of IPF.^[5–8]^ However, the exact causal relationship between lipid metabolites and IPF is still not fully understood.

Several investigations in the field of human metabolomics have established a correlation between lipid metabolites and IPF. However, it is worth mentioning that these associations may conflict with prospective findings in different study cohorts. Serum metabolomics analyses were conducted on 2 observational cohorts of IPF patients from Germany (n = 122), Spain (n = 21), and healthy controls (n = 16), revealed a down-regulation of lysophosphatidylcholine and phosphatidylcholine in the serum of IPF patients.^[9]^ Interestingly, 2 other independent studies on IPF populations (Cohort 1: IPF n = 10, healthy population n = 10; Cohort 2: IPF n = 11, and healthy individuals n = 10) reported contrasting outcomes.^[10]^ Additionally, the dysregulation of PGE2 has been implicated in the development of IPF.^[11]^ However, a study by Li et al found no significant difference in serum PGE2 concentrations between IPF patients and healthy volunteers.^[12]^ Possible factors contributing to the disparate experimental outcomes include confounding variables within the observed population, discrepancies in detection methodologies, and the influence of reverse causality, among others.^[13]^ Therefore, it is crucial to urgently identify biomarkers that demonstrate a strong causal relationship with IPF, instead of solely relying on mutually exclusive observations.

Mendelian randomization (MR) is based on the use of single-nucleotide polymorphisms (SNPs) that are associated with genetic variation as instrumental variables (IVs).^[14,15]^ MR is advantageous in avoiding confounding factors associated with sampling compared to randomized controlled trials.^[16]^ It is also cost-effective, efficient, and increasingly preferred for determining causality between exposure and disease. Therefore, this study aimed to explore potential risk factors related to the association between fatty acid metabolites and IPF using a two-sample MR framework.

## 2. Materials and methods

### 2.1. Bidirectional multivariable two-sample Mendelian randomized design

**Figure 1.**
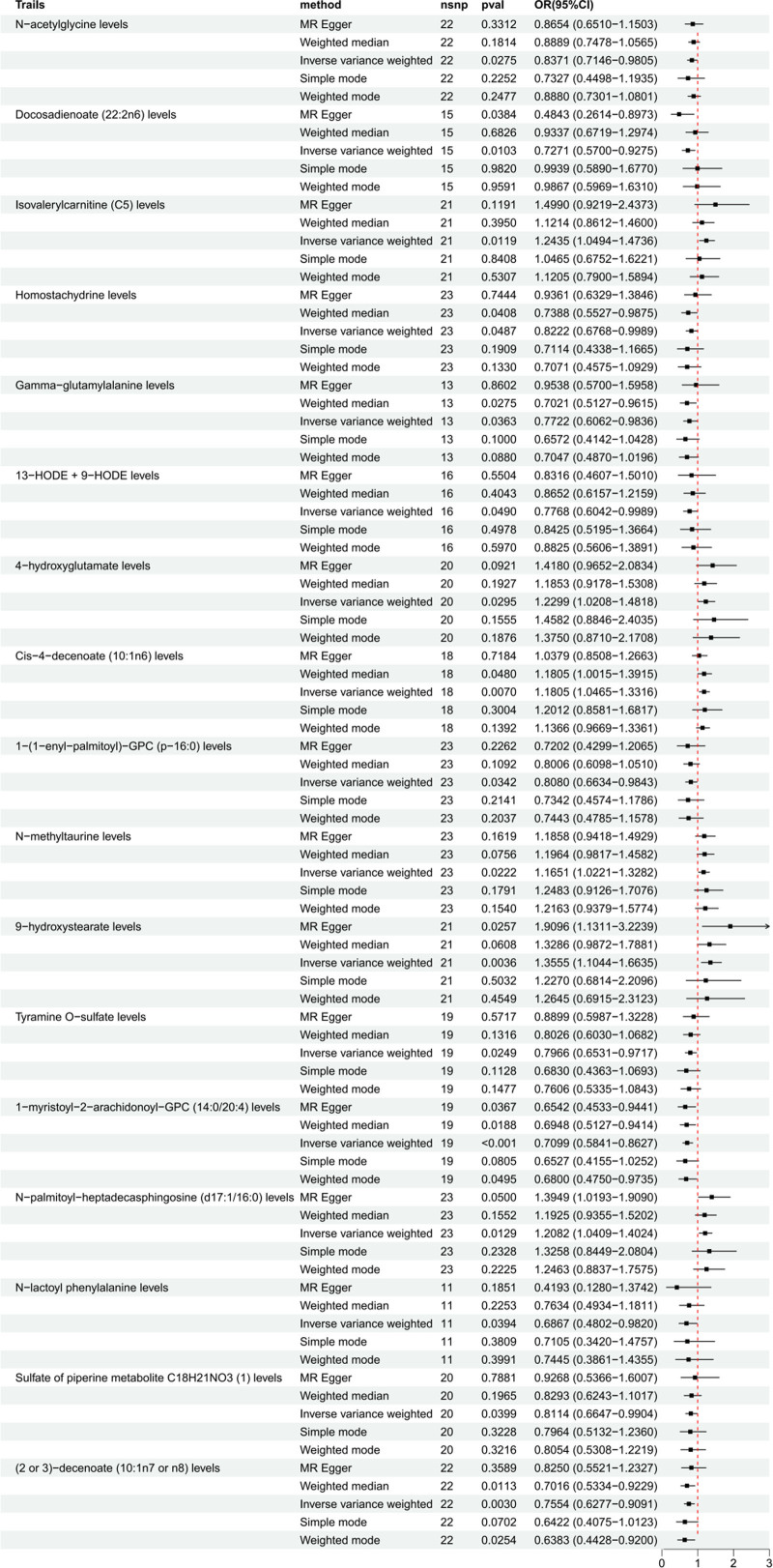
Under the two-sample MR framework, forest plots were generated using 1400 plasma metabolites as the exposure variable and IPF as the outcome variable. The primary method employed was IVW. PIVW < 0.05 indicates a statistically significant causal relationship between metabolites and IPF. The strength of the association between exposure and disease is quantified by the OR and its corresponding 95% confidence interval. IPF = idiopathic pulmonary fibrosis.

Multivariate MR is a valuable method for assessing the causal effect of multiple exposures on outcomes.^[17]^ The method relies on 3 fundamental assumptions: (A) the hypothesis of correlation, which states that there must be a correlation between genetic IVs and the exposure; (B) the hypothesis of independence, which suggests that these IVs should not be associated with any confounding factors; and (C) the hypothesis of exclusion, which asserts that the effects of IVs on the results are solely due to their impact on exposure to a specific focal point, without considering the influence of any other restricting factors. This allows for the deduction of the causal effect of the exposure on the disease.^[18]^ The analysis model of this study is illustrated in Figure 1.

### 2.2. Data source

To investigate the relationship between blood fatty acid metabolites and IPF, we utilized a dataset from the Canadian Longitudinal Study of Aging cohort. The dataset consisted of 1091 metabolites and 309 metabolite ratios obtained from the blood of 8299 individuals. The original experiment was conducted with the approval of the Ethics boards from the Jewish General Hospital (protocol number 2021-2762).^[19]^ The genome-wide association study (GWAS) summary included a total of 1,048,575 SNPs from 1812 IPF patients and 338,784 controls of European ancestry were as outcome. SNPs, the ethics review board of the Hospital District of Helsinki and Uusima approved the FinnGen study protocol. It is important to note that all the populations analyzed in the GWAS were of European descent. The GWAS data was obtained from the UK Biobank cohort and the FinnGen consortium R8 (Tables S1 and S2, Supplemental Digital Content, https://links.lww.com/MD/P560).

### 2.3. Instrumental variable

To meet the requirements of IVs, only SNPs with a *P*val < 1 × 10^−5^ for both exposure and outcome were included in the statistical analysis.^[20]^ Clump, an important feature in PLINK software (version 1.90), was used to cluster, and analyze genetic data.^[21]^ It was employed to remove data linkage imbalances and redundant SNPs, based on a linkage disequilibrium (LD) threshold of *R*^2^ < 0.001 within a 10,000 kb distance. Specifically, the closer the *R*^2^ value is to 0, the higher the degree of complete linkage balance between the 2 SNPs. Rejecting all other SNPs within 10,000 kb of a given SNP ensures independence. Additionally, any SNP with an *F* statistic value > 10 was considered a strong variable and included in the analysis. After determining the association between SNPs and IPF, we controlled for confounding factors such as smoking, type 2 diabetes, and gastro-esophageal reflux.^[22–25]^

### 2.4. Mendelian randomization

Bidirectional, two-sample MR studies have provided evidence for a causal relationship between blood metabolites and IPF. We used the R package “TwoSampleMR” (Version R4.3.2) to identify alleles that are significantly related. To ensure unidirectional causality, we employed 5 statistical approaches.^[26]^ Inverse variance weighted method (IVW), the primary method, demonstrated a crucial causal association. An odds ratio (OR) < 1 indicates increased risk, while an OR > 1 indicates reduced risk. PIVW < 0.05 indicates statistical significance between exposure IV and outcome IV.^[27]^ Additionally, we used 4 auxiliary discrimination methods that required the OR value to be consistent with the IVW OR. These methods include the MR-Egger method, which utilizes Egger regression to estimate the causal effect.^[28]^ The simple mode method, which provides quick results. The weighted median method, which calculates the median of the data,^[29]^ and the weighted pattern method, which calculates the mode (i.e., the value that occurs most frequently) of the data. All relevant analyses were based on the Ensemble GRCh38 reference for genome coordinates.^[30]^

### 2.5. Multiple sensitivity analysis

To address the balanced polytropy of the MR hypothesis, a robustness analysis was conducted.^[31]^ Firstly, the MR-Egger intercept method was employed to assess horizontal pleiotropy resulting from genetic variation influenced by multiple traits. The aim was to mitigate bias in the findings, with a *P*val > .05 indicating no confounding factors.^[32]^ Secondly, the Cochran *Q* test was executed under the IVW method and MR-Egger method to check for heterogeneity across the individual causal effects. A *Q*-*p*val > .05 indicates no heterogeneity level.^[33]^ Finally, the leave-one-out method was used to test the sensitivity of the model in each iteration of the dataset, ruling out the possibility of summary results being driven by a single abnormal SNP. The value > 0 for all iterations indicates that the result is reliable.^[34]^

## 3. Results

### 3.1. Instrument strength

We utilized the most up to date GWAS data, which included information on 1400 blood metabolites. To ensure accuracy, we removed any linkage disequilibrium. For a strong IV, we set a criterion of *F*-statistic values > 10. All SNPs that met this criterion can be found in the supplementary materials (Table S3, Supplemental Digital Content, https://links.lww.com/MD/P560 displays the SNPs associated with major metabolite results). Additionally, we identified a total of 31 SNPs as IVs for IPF genetic variables, which is considered sufficient for conducting MR analysis (Table S4, Supplemental Digital Content, https://links.lww.com/MD/P560 provides further details).

### 3.2. Causal relationship between blood metabolites and IPF

**Figure 2.**
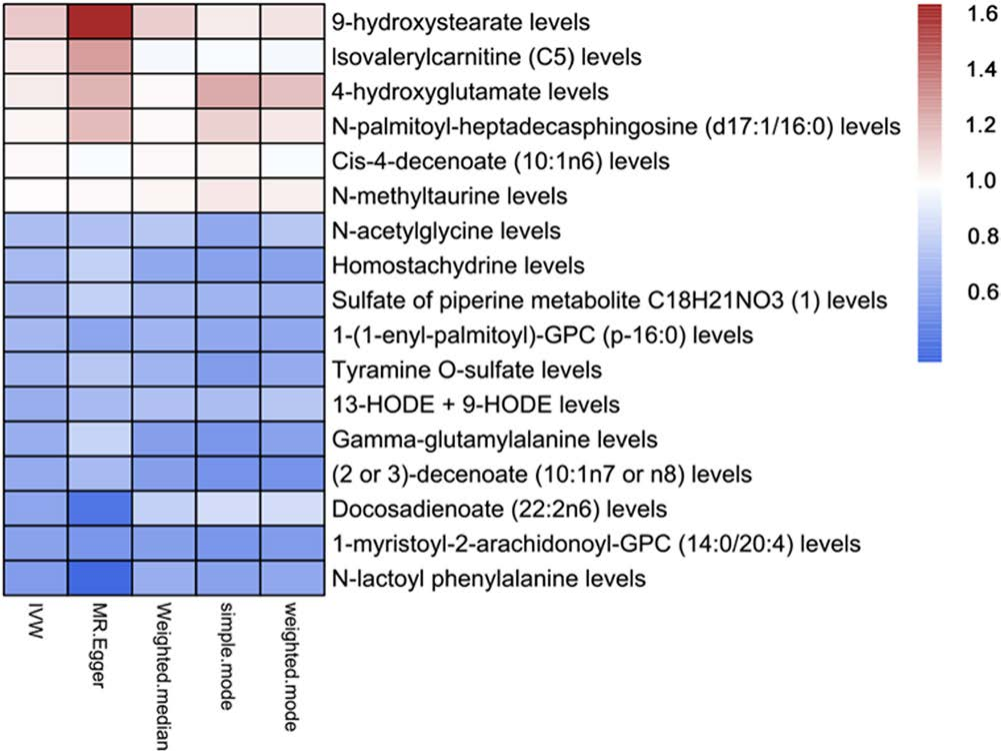
Heat maps were drawn according to OR values of 5 methods to reflect the degree of protection and risk of exposure to IPF. IPF = idiopathic pulmonary fibrosis.

Through rigorous quality control measures, particularly the IVW method, we identified 17 plasma metabolites that showed significant causal associations with IPF (Fig. 2).

**Figure 3.**
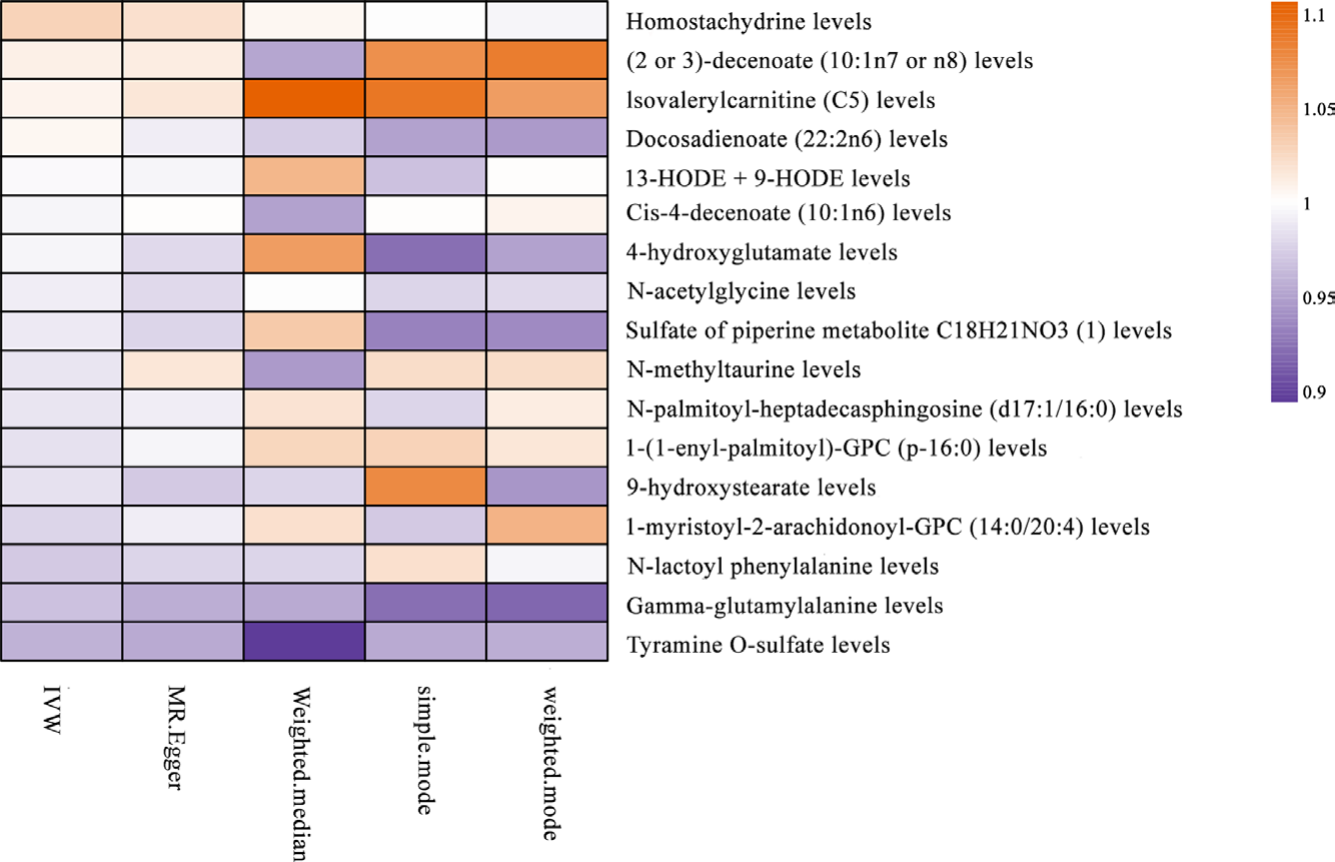
Heat maps were drawn based on the OR values of the 5 methods to reflect the strength of the association between IPF and 17 candidate metabolites under different methods.

These metabolites consisted of lipid metabolites (9/17), amino acid metabolites (5/17), alkaloids (2/17), and amine metabolites (1/17). The beta values obtained from different methods for all indicators exhibited good consistency, highlighting the robustness of the observed risk or protection across various test methods (Figure S1, Supplemental Digital Content, https://links.lww.com/MD/P561). Our findings revealed that 6 metabolites were positively correlated with IPF, while 11 metabolites were negatively correlated (Fig. 3 and Table S5, Supplemental Digital Content, https://links.lww.com/MD/P560).

Notably, various metabolites, such as fatty acids, lipid metabolites, lipid peroxides, and phospholipids, are involved in lipid metabolic pathways. This provides clear evidence of the involvement of fatty acid metabolites in IPF, which aligns with the lipid changes primarily observed in patients with IPF^[9]^ (Table 1).

**Table 1:** Nine lipid metabolites that are causally associated with IPF.

Outcome	Metabolite name	Metabolite class
IPF	9-Hydroxystearate	Fatty acid
	lsovaleryicarnitine (C5)	Fatty acyls
	N-palmitloyl-heptadecasphingosine (d17:1/16.0)	Sphingolipid
	Cis-4-decenoate (10:1n6)	Fatty acid
	1-(1-Enyl-palmiloyl-GPC (*P*-16:0)	Phospholipids
	Docosadienoate (22 2n6)	Fatty acid
	1-Myristoyl-2-arachidonoyl-GPC (14:0120:4)	Phospholipids
	13-HODE + 9-HODE	Lipid peroxide
	(2 or 3)-Decenoale (10:1n7 or n8)	Fatty aldehydes

IPF = idiopathic pulmonary fibrosis.

### 3.3. Reverse MR analysis of IPF associated with 17 candidate metabolites risk

**Figure 4.**
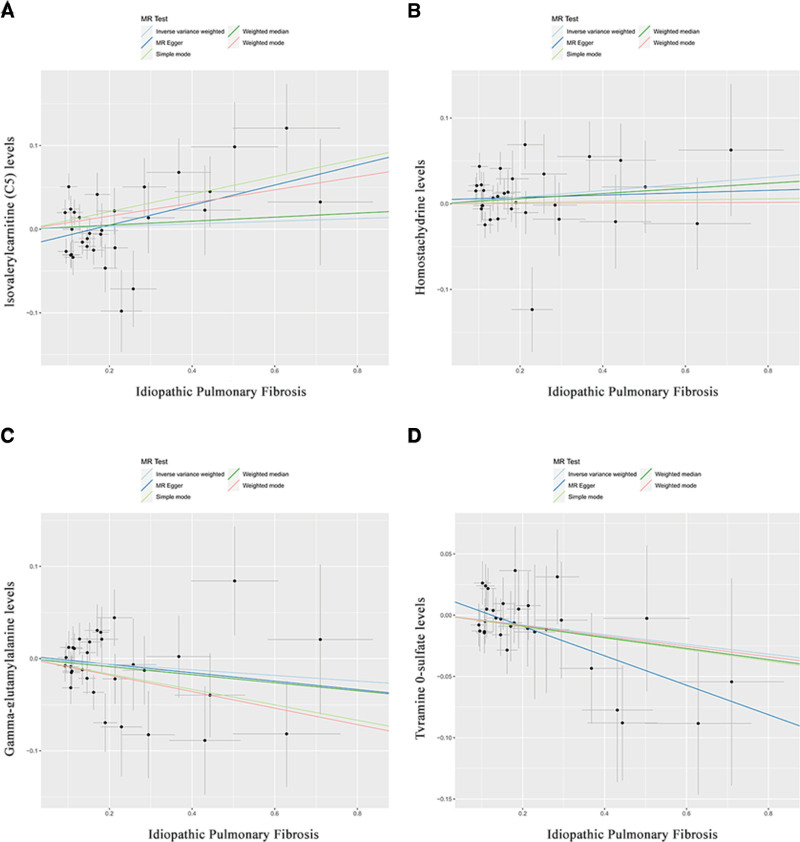
Scatter plot based on 5 tests (beta value in the same direction) in reverse Mendelian randomization analysis. There was no significant causal relationship between IPF and the 4 plasma metabolites, including (A) lsovaleryicarnitine C5 levels; (B) Homostachydrine levels; (C) Gamma-glutamytalanine levels; (D) Tyramine O-sulfate levels.

To further explore the relationship between IPF and candidate metabolites, we conducted reverse analysis. In this analysis, IPF was considered as the exposure variable and the 17 blood metabolites as the outcome variables. Using the IVW method, we found that IPF did not significantly contribute to abnormal changes in the 17 metabolites (PIVW > 0.05, Table S6, Supplemental Digital Content, https://links.lww.com/MD/P560). We also assessed the consistency of OR and beta values obtained from 5 different statistical methods to determine the direction, intensity, and probability of IPF’s influence on the candidate metabolites (Fig. 4 and Table S6, Supplemental Digital Content, https://links.lww.com/MD/P560).

**Figure 5.**
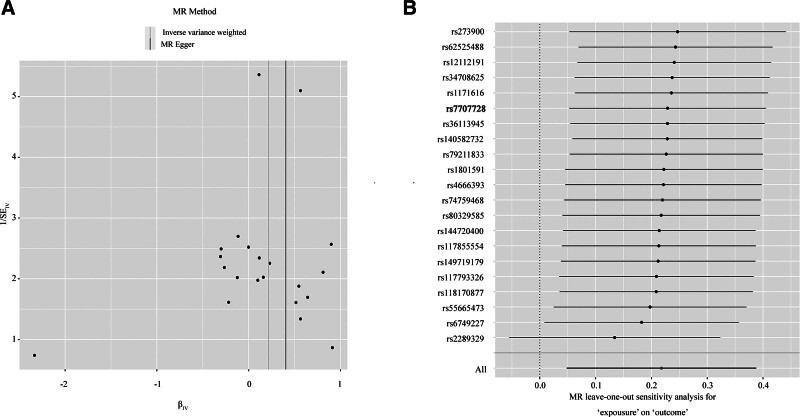
Visualizes two-sample MR analysis of IPF and Isovallerylcarnitine (C5) levels level pleiotropy and sensitivity analysis through funnel plots and leave-one-out plots, respectively. (A) Funnel plot of and (B) leave-one-out permutation analysis plot of IPF.

Through this analysis, we excluded 13 metabolites (Figure S2, Supplemental Digital Content, https://links.lww.com/MD/P561) and identified a noncausal relationship between IPF and 4 candidate blood metabolites (Table 2): Isovalerylcarnitine (C5) levels (Fig. 5A), Homostachydrine levels (Fig. 5B), Gamma-glutamylalanine levels (Fig. 5C), and Tyramine O-sulfate levels (Fig. 5D).

**Table 2:** Reverse MR analysis results.

Exposure outcome	Method	PIVW	OR (95% CI)
IPF Isovalerylcarnitine (C5) levels	Inverse variance weighted	0.6253	1.0159 (0.9533–1.0826)
IPF Homostachydrine levels	Inverse variance weighted	0.1371	1.0392 (0.9877–1.0934)
IPF Gamma-glutamylalanine levels	Inverse variance weighted	0.2638	0.9700 (0.9195–1.0232)
IPF Tyramine O-sulfate levels	Inverse variance weighted	0.1101	0.9610 (0.9154–1.0090)

IPF = idiopathic pulmonary fibrosis, IVW = inverse variance weighting method, MR = Mendelian randomization, OR = odds ratio.

Statistical significance was defined as *P* < .05.

#### 3.3.1. The sensitivity analysis shows that Isovalerylcarnitine (C5) levels is a good index to predict IPF

**Figure 6.**
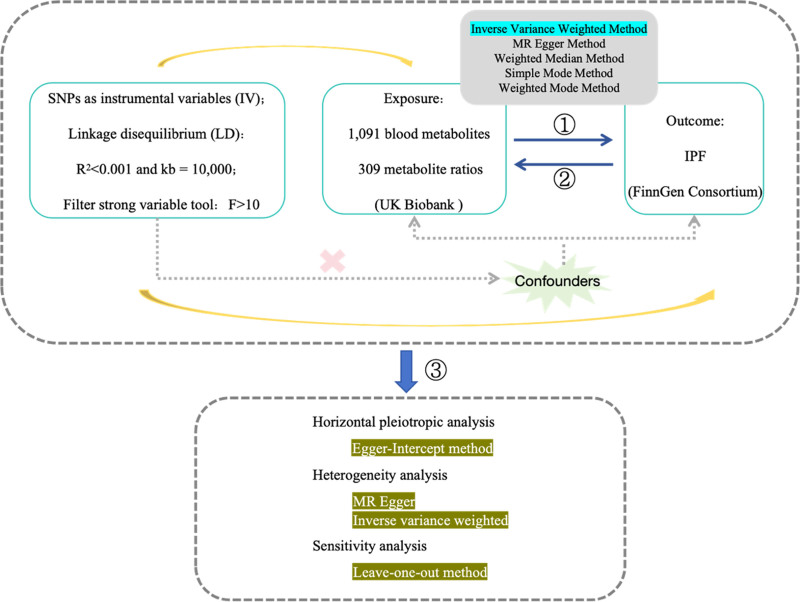
Schematic representation of the two-sample MR hypothesis. The MR analysis investigates the association between plasma metabolite levels and IPF risk. LD denotes the nonrandom association of alleles at different loci; *R*^2^ represents the squared correlation coefficient; *F* > 10 indicates a strong IV; The inverse variance weighted method is the primary causal determination method, highlighted in blue.

It has been reported that the accumulation of Isovalerylcarnitine (C5) levels leads to mitochondrial dysfunction and impaired oxidation of fatty acid β in pediatric drug-resistant epilepsy,^[35]^ while mitochondrial dysfunction and metabolic reprogramming occur frequently in IPF lung.^[36]^ Therefore, we speculate whether Isovalerylcarnitine (C5) levels is also the originator of mitochondrial dysfunction in IPF patients, which is exactly consistent with our results. Increased Isovalerylcarnitine (C5) levels is a risk factor for IPF. Next, we diligently carried out horizontal pleiotropy analysis, heterogeneity analysis, and sensitivity analysis to effectively address estimation bias caused by pleiotropy of genetic variation in causal analysis utilizing MR. Egger Intercept showed that horizontal pleiotropy does not exist in our analysis process (β = ‐0.027; SE = 0.0337; *P*val = .4310). The MR-egger and IVW methods suggested that there is no heterogeneity in this experiment (MR-egger *Q*-*p*val = .4439; IVW *Q*-*p*val = .4669) (Fig. 6A and Table S7, Supplemental Digital Content, https://links.lww.com/MD/P560). Furthermore, the leave-one-out method reveals that causal effects do not exist driven by a single IV (Fig. 6B and Table S8, Supplemental Digital Content, https://links.lww.com/MD/P560). In short, comprehensive analysis showed that our results have good predictive value.

## 4. Discussion

Patients with IPF exhibit dysregulation of blood metabolism. However, the precise regulatory mechanism underlying the relationship between IPF occurrence and changes in metabolites remains unclear. In this pioneering study, our objective was to investigate the causal relationship between blood metabolites and IPF using a bidirectional, multivariable two-sample MR approach. To achieve this, we utilized the largest publicly available GWAS dataset, comprising 1400 blood metabolites, to identify robust genetic variants associated with IPF. Through comprehensive genetic analysis of over 340,000 Europeans, we discovered that genetic susceptibility to 4 certain blood metabolites is causally linked to IPF. Specifically, this study revealed that Isovalerylcarnitine (C5) levels is a risk factor for IPF via rigorous bidirectional MR testing and sensitivity analysis.

Isovalerylcarnitine (C5) levels plays a role in the metabolic oxidation of branchchain fatty acids in mitochondria.^[37]^ A previous study has suggested that ramipril’s positive health effects on SARS-CoV-2 might be attributed to its ability to reduce plasma Isovalerylcarnitine (C5) levels, in other words, an increase in Isovalerylcarnitine (C5) levels s in SARS-CoV-2 could potentially interfere with normal lung function.^[38]^ Elevated concentrations of Isovalerylcarnitine (C5) levels have been observed in individuals with obesity, type 2 diabetes, and plasma ND1 mtDNA ≥ 3200 copies/μL. suggested a potential association between Isovalerylcarnitine (C5) levels and mitochondrial dysfunction.^[39–41]^ It has been demonstrated that changes in lipid metabolism can strongly contribute to mitochondrial dysfunction,^[42]^ which is known to be a driving factor in IPF.^[43,44]^ Impaired fatty acid β-oxidation can also result from mitochondrial dysfunction,^[45]^ De Perrot et al found that the key enzymes and metabolites of the mitochondrial β-oxidation pathway in the lungs of IPF patients are altered.^[46]^ However, there is currently no clear study investigating whether the disorder of lipid metabolism triggers IPF or if lipid metabolism is altered after the onset of IPF. Our results provide a potential pathogenic biological factor for IPF. We speculate that the increase in Isovalerylcarnitine (C5) levels in serum may lead to lipid deposition in lung cells and disrupt mitochondrial function, thereby increasing the risk of IPF. However, this hypothesis needs to be further validated through a large prospective cohort study.

In pulmonary fibrosis, metabolomic abnormalities in various cell types, including cancer-associated fibroblasts (CAFs),^[47]^ alveolar epithelial cells,^[48]^ and macrophages,^[49]^ contribute to abnormal collagen synthesis and dysregulated airway remodeling.^[50]^ Studies indicate that abnormal levels of Isovalerylcarnitine (C5) may influence the onset and progression of IPF through multiple signaling pathways. Recently, newborn screening in Wisconsin revealed significantly elevated Isovalerylcarnitine (C5) levels in children with short/branched-chain acyl-CoA dehydrogenase deficiency, and the exon of the ACADSB gene was found to be active in this population.^[51]^ ACADSB is enriched in the fatty acid metabolic pathway of tumor cells, and an increase in its expression level may promote the generation of acetyl-CoA. The accumulation of acetyl-CoA further influences the lipid metabolism of CAFs by reprogramming the CXCL5–CXCR2 axis.^[52]^ It is therefore speculated that the increased risk of IPF associated with Isovalerylcarnitine (C5) may be attributed to the disruption of lipid metabolism in CAFs through the regulation of key genes in the lipid metabolism pathway. Interestingly, in CNS encephalitis, the abnormal elevation of Isovalerylcarnitine (C5) levels and the increase in lactate concentration suggest that elevated extracellular levels of acylcarnitine may interfere with the β-oxidation of saturated fatty acids in mitochondria.^[53]^ Consequently, cells may resort to glycolysis for energy, leading to the extrusion of acylcarnitine complexes into the cytoplasm and their subsequent expulsion into the extracellular environment.^[54]^ Research indicates that high levels of acylcarnitine in cardiac tissue can disrupt biological membranes through nonspecific detergent effects, induce electrophysiological changes, and inhibit several key enzyme systems.^[55]^ Whether elevated Isovalerylcarnitine (C5) levels affect cellular homeostasis in alveolar epithelial cells remains an open question, representing an intriguing hypothesis. Existing evidence indicates that the detrimental effects of long-chain acylcarnitines manifest during reperfusion, with mitochondria damaged by acylcarnitines inevitably generating reactive oxygen species (ROS) when oxygen supply is restored.^[54]^ The accumulation of ROS is a hallmark of IPF, and elevated ROS levels facilitate the translocation of P65 to the nucleus, resulting in the upregulation of markers associated with pulmonary fibrosis.^[56]^ Furthermore, ROS can promote the inactivation of lung epithelial cells and propel the progression of pulmonary fibrosis through various mechanisms, including inflammation, oxidative stress, and alterations in glucose metabolism.^[57]^ These findings elucidate the intricate interplay between fatty acid metabolites and signaling pathways in the context of IPF.

Statistics on the incidence of IPF vary widely between health systems.^[58]^ This variability may be attributed to the increasing awareness and diagnosis of the disease. Currently, the diagnosis of IPF is invasive, such as collecting BAL fluid and freezing lung biopsies, which may increase the risk of disease progression. The latest ATS/ERS/ALAT/JRS guidelines have updated the diagnostic algorithm for IPF,^[59]^ suggesting the use of serological assisted diagnosis. However, serological tests currently lack specific diagnostic markers and need to rule out a variety of diseases that may cause ILD. Therefore, it is important to conduct reliable and convenient biomarker studies for IPF. Qian et al identified differentially expressed transcripts involved in lipid metabolism in IPF lung tissue,^[60]^ while Yin Lyu et al identified 5 fatty acid metabolite-related genes in bronchoalveolar lavage fluid of IPF patients.^[61]^ Additionally, Miriana d’Alessandro found differential lipid metabolites in IPF alveolar lavage fluid, however, these lipid metabolites do not possess diagnostic value in serum for distinguishing IPF from hypersensitivity pneumonitis.^[62]^ The strong association between IPF and lipid metabolism drove us to look for potential associations between lipid metabolites and IPF by examining blood metabolites. The evidence provided by MR in our study indicates a causal relationship between Isovalerylcarnitine (C5) levels and IPF, with no reverse causality observed. Therefore, increased levels of Isovalerylcarnitine (C5) levels may increase susceptibility to IPF, suggesting its potential as a candidate biomarker for IPF diagnosis. However, further validation from clinical samples is necessary to determine whether Isovalerylcarnitine (C5) levels can be utilized as a diagnostic indicator of IPF.

Limitations of this study: Firstly, due to the short median survival time of 2 to 3 years after IPF diagnosis and the absence of typical process nodes during the stable period, decline period, and rapid deterioration of the disease,^[63]^ we were unable to obtain typical GWAS data for IPF at each stage. Therefore, we cannot discuss the causal relationship between Isovalerylcarnitine (C5) levels and the IPF process. Secondly, since many serum biomarker levels are continuous variables, further measurements, and statistical analysis of causal dichotogenic data are necessary to determine thresholds corresponding to significant abnormalities. Thirdly, it is important to note that our data population consists solely of individuals of European descent. Although the GWAS of exposure and outcome are derived from different public databases, the two-sample MR analysis is often affected by sample overlap, which may limit the causal relationship derived by MR in terms of race selection.^[64]^ Therefore, it is essential to explore IPF GWAS data in diverse populations to achieve a more comprehensive understanding of the disease. Standardized measurements of Isovalerylcarnitine (C5) and the pulmonary fibrosis model would be employed to address the same issue. Future studies must confirm potential mechanisms at the RNA, protein, and metabolic levels, both in vitro and in vivo, while including a larger collection of IPF samples.

## 5. Conclusion

Our study utilized publicly published GWAS data to evaluate the random effect of blood metabolites on IPF using a two-way two-sample MR Approach. Specifically, our bidirectional MR Analysis revealed a unidirectional causal relationship between Isovalerylcarnitine (C5) levels and IPF, indicating that Isovalerylcarnitine (C5) levels serves as a risk factor for IPF.

## Author contributions

**Conceptualization:** Sijing Liu.

**Data curation:** Zhengyue Liao.

**Formal analysis:** Jing He, Zhengyue Liao.

**Funding acquisition:** Jinlin Guo.

**Investigation:** Jing He, Hongyu Chen.

**Methodology:** Hongyu Chen, Jinlin Guo.

**Software:** Jiaojiao Fu.

**Supervision:** Sijing Liu, Ya’nan Hua.

**Validation:** Sijing Liu, Ya’nan Hua, Jinlin Guo.

## Supplementary Material


