# Supplementary material for: Mendelian randomization revealed a one-way causal association between increased Isovalerylcarnitine (C5) levels and the risk of idiopathic pulmonary fibrosis
Source: Medicine (Baltimore). 2025 Aug 8;104(32):e43555. doi: 10.1097/MD.0000000000043555 (PMC12338245; doi:10.1097/MD.0000000000043555)
Supplement: Supplementary file 2 [file medi-104-e43555-s002.docx]

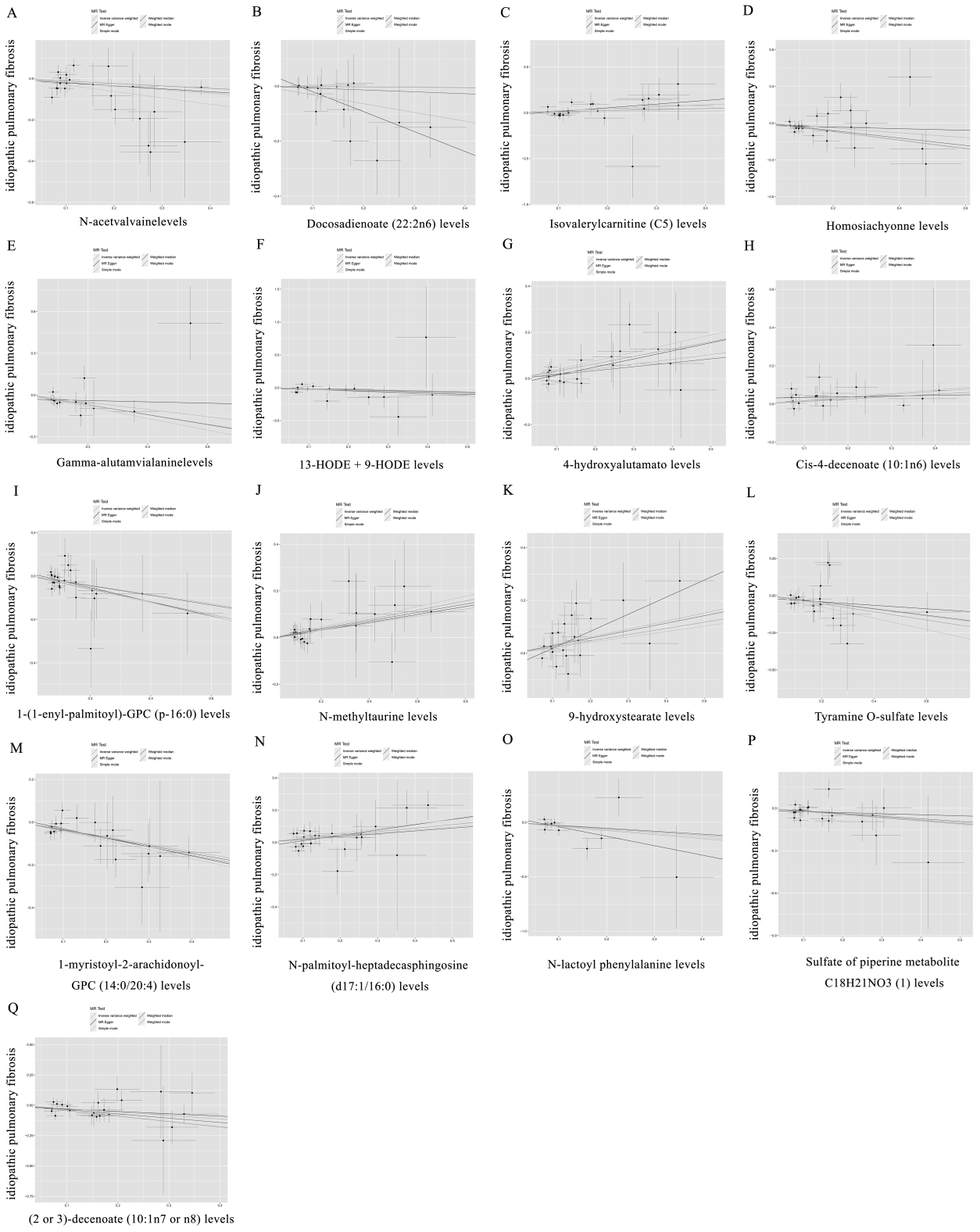


**Figure S1. Scatter plot based on five tests in Forward mendelian randomization analysis.**

(A) N-acetylglycine levels; (B) Docosadienoate (22:2n6) levels;

1. lsovalerylcarnitine (C5) levels; (D) Homostachydrine levels; (E) Gamma-glutamylalanine levels; (F)13-HODE + 9-HODE levels; (G) 4-hydroxyglutamate levels; (H) Cis-4-decenoate (10:1n6) levels; (I) 1-(1-enyl-palmitoyl)-GPC (p-16:0) levels;

(J) N-methyltaurine levels; (K) 9-hydroxystearate levels; (L) Tyramine O-sulfate levels; (M) 1-myristoyl-2-arachidonoyl-GPC (14:0/20:4) levels; (N) N-palmitoyl-heptadecasphingosine (d17:1/16:0) levels; (O) N-lactoyl phenylalanine levels; (P) Sulfate of piperine metabolite C18H21NO3 (1) levels; (Q) (2 or 3)-decenoate (10:1n7 or n8) levels.


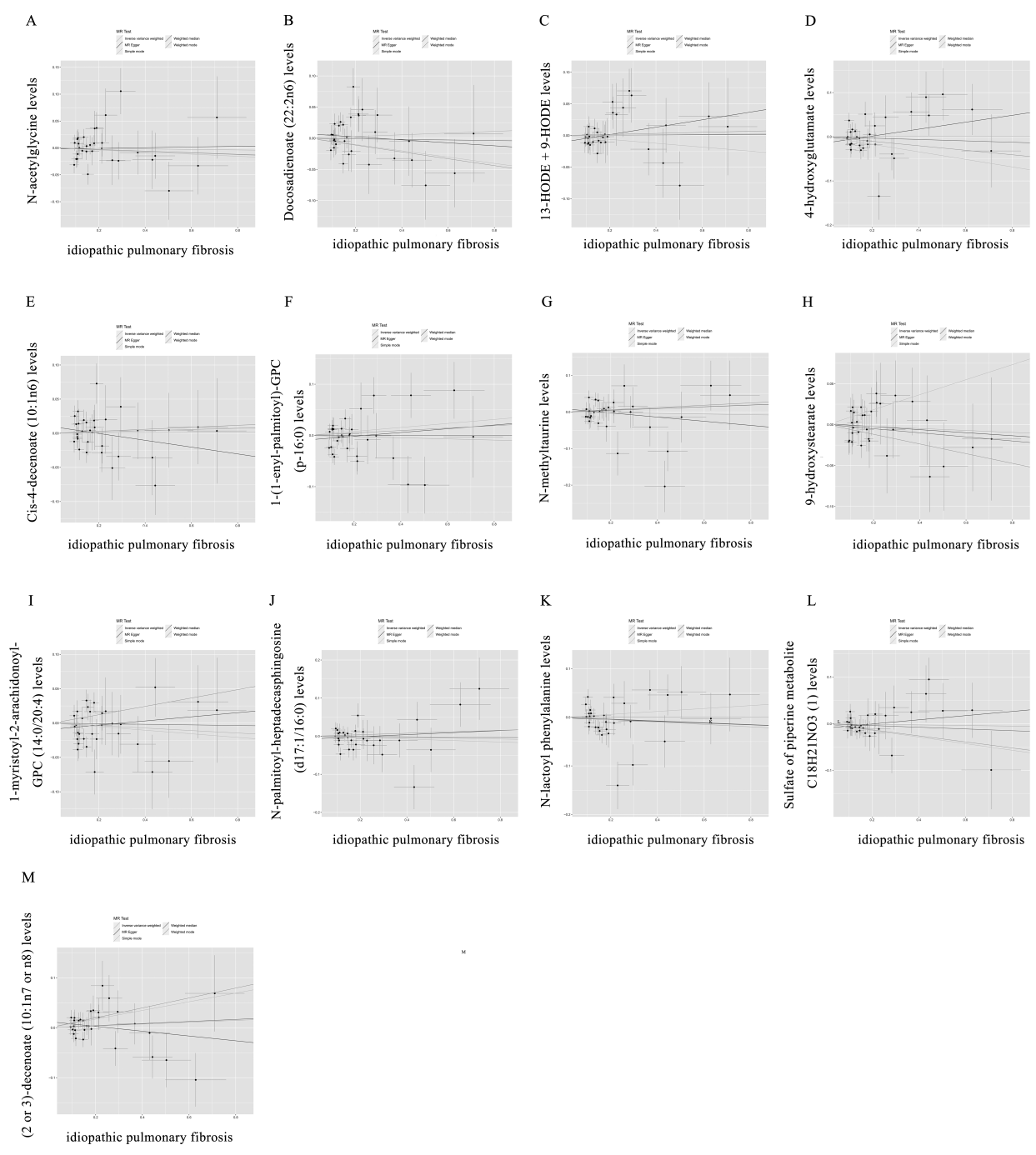


**Figure S2. Scatter plot based on five tests in reverse mendelian randomization analysis.** (A) N-acetylglycine levels; (B) Docosadienoate (22:2n6) levels;

(C)13-HODE + 9-HODE levels; (D) 4-hydroxyglutamate levels; (E) Cis-4-decenoate (10:1n6) levels; (F) 1-(1-enyl-palmitoyl)-GPC (p-16:0) levels; (G) N-methyltaurine levels;

1. 9-hydroxystearate levels; (I) 1-myristoyl-2-arachidonoyl-GPC (14:0/20:4) levels;
2. N-palmitoyl-heptadecasphingosine (d17:1/16:0) levels; (K) N-lactoyl phenylalanine levels; (L) Sulfate of piperine metabolite C18H21NO3 (1) levels; (M) (2 or 3)-decenoate (10:1n7 or n8) levels.
